# Molecular Systematics of the Phoxinin Genus *Pteronotropis* (Otophysi: Cypriniformes)

**DOI:** 10.1155/2015/298658

**Published:** 2015-05-31

**Authors:** Richard L. Mayden, Jason S. Allen

**Affiliations:** ^1^Department of Biology, Saint Louis University, 3507 Laclede Avenue, St. Louis, MO 63103, USA; ^2^Department of Biology, Saint Louis Community College, Meramec Campus, 11333 Big Bend Road, St. Louis, MO 63122, USA

## Abstract

The genus *Pteronotropis* is widely distributed along the gulf slope of eastern North America from Louisiana to Florida and rivers in South Carolina along the Atlantic slope. *Pteronotropis* have very distinctive, flamboyant coloration. The habitats most frequently associated with these species include heavily vegetated backwater bayous to small sluggish or flowing tannin-stained streams. Although *Pteronotropis* is recognized as a valid genus, no phylogenetic analysis of all the species has corroborated its monophyly. In recent years, four additional species have been either described or elevated from synonymy: *P. merlini*, *P. grandipinnis*, *P. stonei*, and *P. metallicus*, with the wide-ranging *P. hypselopterus* complex. To examine relationships within this genus and test its monophyly, phylogenetic analyses were conducted using two nuclear genes, recombination activating gene 1, RAG1, and the first intron of S7 ribosomal protein gene in both maximum parsimony and Bayesian analyses. In no analysis was *Pteronotropis*, as currently recognized, recovered as monophyletic without the inclusion of the currently recognized *Notropis harperi*, herein referred to as *Pteronotropis*. Two major clades are supported: one inclusive of *P. hubbsi*, *P. welaka*, and *P. harperi* and the second inclusive of *P. signipinnis*, *P. grandipinnis*, *P. hypselopterus* plus *P. merlini* sister to *P. euryzonus*, and *P. metallicus* plus *P. stonei*.

## 1. Introduction

The subfamily (or family) Leuciscinae includes all cyprinid species in North America, except* Notemigonus*, and species across Eurasia. Many of the species of this North American fauna have been examined in different phylogenetic studies at varying degrees of universality using both morphological and molecular data. Initial morphological studies by Mayden [1] and Coburn and Cavender [2] revealed exciting new relationships and a reclassification of the North American fauna. These studies were followed with several molecular analyses of different major lineages, genera, and species groups that supported many, but not all, of the monophyletic groups previously identified in one or both of the above studies [3–7]. However, not all proposed genera have been examined for species relationships using molecular markers.

One such genus in North America with an increasing and intriguing diversity, biology, and geographic distribution, as well as complex taxonomic history, is* Pteronotropis*. This genus contains one of North America's most colorful shiners.* Pteronotropis hubbsi* and* P. welaka* are relatively slender-body species that, in breeding males, possess enlarged dorsal fins, whereas the remaining species,* P. euryzonus*,* P. hypselopterus*,* P. merlini*,* P. grandipinnis*,* P. stonei*,* P. metallicus*, and* P. signipinnis*, are more deep-bodied and lack enlarged dorsal fins in breeding males. The most frequently associated habitat of these species across their ranges includes deep, backwater bayous, small sluggish tannin-stained streams, and flowing tannin-stained streams, all with ample aquatic vegetation. However, despite several studies on shiners and relatives to date,* Pteronotropis* has received essentially no recent attention as to their relationships and has been proposed to be an unnatural grouping. Herein, we provide the first examination of phylogenetic relationships of all species in the genus (formerly subgenus of* Notropis* [1]) and a test of the monophyly of this purported lineage. Two nuclear genes are used in this analysis because of their previously demonstrated genetic distances and resulting ability to resolve nodes deeper than at the crown of trees. These genes have been used successfully for resolution of more basal lineages of North American cyprinids by several recent papers [3–10].

Resulting phylogenetic inferences of species of this group and their eventual placement relative to other North American cyprinids are critical as they largely facilitate more process-level questions as to the evolution of the biology of the species and other lineages to better understand the processes of anagenesis and speciation. While multiple papers listed above have made groundbreaking strides in providing a phylogenetic framework where one previously did not exist for North American cyprinids, Hollingsworth et al. [10] provide an excellent evaluation of a subset of the fauna and a novel hypothesis as to habitat shifts for clades with differing rates of speciation. Given that no study has examined all of the species of* Pteronotropis*, we provide a review of the history of the genus and molecular phylogenetic analyses of the species using two nuclear genes that result in identical species relationships based on mitochondrial genes in Mayden and Allen [11].

*Taxonomic History*.* Pteronotropis* currently includes nine species in rivers and streams distributed along the gulf slope from Louisiana to Florida and along the Atlantic slope as far north as South Carolina. One species,* P. hubbsi*, currently occurs only in southern Arkansas and northern Louisiana but was likely to be more widely distributed in lowland habitats; the conservation status of this species is of concern, as it has not been found in some locations (including southern Illinois) for several decades.

In a study focusing on 566 morphological traits of a large number of cyprinids, Mayden [1] elevated* Pteronotropis* to generic level and included* P. welaka*,* P. signipinnis*,* P. hypselopterus*, and* P. euryzonus* within the genus but left* P. hubbsi* in* Notropis*. Recently, Suttkus and Mettee [12], with no characters, phylogenetic analysis, or substantive phylogenetic argument, maintained that* Pteronotropis* was a subgenus within the genus* Notropis* (as classified before Mayden's [1] analysis) and that this subgenus contained only* P. euryzonus* and the* P. hypselopterus* complex (*P. hypselopterus*,* P. grandipinnis*,* P. stonei*,* P. metallicus*, and* P. merlini*).

The phylogenetic relationships of* Pteronotropis* have been somewhat enigmatic over the years. Species share derived and distinctive color patterns that include bright red-orange to yellow striped dorsal, caudal, and anal fins and a broad dark lateral band extending from the head to the caudal peduncle. The genus was divided into two groups based on morphological and molecular characters [1, 4].* Pteronotropis signipinnis* was described by Bailey and Suttkus [13] and was considered a member of the genus* Notropis* (subgenus* Pteronotropis* by Fowler [14]), along with* P. hypselopterus*.* Pteronotropis euryzonus* [15] was later added to this subgenus and was considered a close relative to* P. hypselopterus*; however, neither of the above two studies included* P. hubbsi* or* P. welaka* and they were conducted in a prephylogenetic era.* Pteronotropis hubbsi* was described by Bailey and Robison [16] and was thought to be closely related to* P. welaka*; at that time, neither species was allocated to the subgenus* Pteronotropis*. In a study utilizing twenty-one allozyme loci, Dimmick [17] examined nine species (mostly* Pteronotropis*). This allozyme analysis revealed* Pteronotropis* as nonmonophyletic, with* P. hubbsi* and* P. welaka* as distantly related and* N. signipinnis* and* N. hypselopterus* as sister species. Consequently, Dimmick [17] argued that all of the morphological characters of Bailey and Robison [16], thought to indicate a close relationship between* P. hubbsi* and* P. welaka*, were the result of convergent evolution.

In the first sequence analysis of this group, Simons et al. [18] used mitochondrial cytochrome* b* gene and failed to corroborate* Pteronotropis* as a monophyletic group. With both parsimony and likelihood analyses,* P. euryzonus* was sister to* P. hypselopterus* and an unrelated clade included* P. signipinnis* sister to* P. hubbsi* plus* P. welaka*. Later, in a subsample of* Pteronotropis* species, Simons et al. [4], using two mitochondrial genes (12S, 16S), and Bufalino and Mayden [5, 6], using two nuclear loci (RAG1, S7), found* Pteronotropis* as monophyletic but, again, only with the inclusion of “*Notropis*”* harperi*; however, neither of these analyses included all species of the genus. Other early molecular data and analyses also failed to resolve the phylogenetic relationships of the above species that were generally phenetically similar. Most recently a study by Hollingsworth et al. [10], using one mtDNA gene and nDNA genes, corroborated the monophyly of a subsample of species of* Pteronotropis* that also included* N. harperi*.

While there have been several efforts testing the monophyly of* Pteronotropis*, its composition, and at resolving the phylogenetic relationships of species since its elevation to genus, no single study has included all of the species in the genus and appropriate outgroups based on earlier studies and some did not include the morphologically similar* Notropis harperi*. With the elevation of species from synonymy with* P. hypselopterus* and the description of a new species [12], the complexity involved in testing the monophyly of the genus and species relationships have become even more biologically interesting. While Suttkus and Mettee [12] did provide dialogue invoking phylogenetic terminology as to species relationships, their study contained no phylogenetic analyses, no discussions of character homology, or any morphological or molecular synapomorphies. To date, no investigation has been completed for this group inclusive of all of the purported species of* Pteronotropis*. Thus, the objectives of the current study are twofold: (1) testing the monophyly of the genus and (2) examining relationships of all of the purported species of the genus using two nuclear genes.

## 2. Materials and Methods

### 2.1. Specimens and DNA Extraction/Amplification and Alignment

Museum catalogue numbers for vouchers in this study include UAIC (University of Alabama Ichthyological Collection) and SLUM (Saint Louis University Museum). Specimens examined in this study were either frozen at Saint Louis University, preserved in 95% ethanol, or captured alive and transported to Saint Louis University (Table 1). Outgroup taxa included species from the genera* Cyprinella*,* Lythrurus*, and* Notropis*. Species of* Cyprinella* were included as outgroup taxa due to previous studies indicating their close relationships to* Pteronotropis*. Because this analysis focuses on nuclear gene variation as it contributes to phylogenetic relationships and the inadequate sampling of all relevant taxa in previous studies, cytochrome* b* sequences of previous mitochondrial analyses are not included. Genomic DNA was extracted using the QIAGEN QIAamp tissue kit according to the manufacturer's recommendations (QIAGEN, Valencia, CA). The two nuclear genes included recombination activating gene 1, RAG1, and the first intron of S7 ribosomal protein gene. Both genes were amplified, via PCR, and internal primers amplification and sequencing were developed for S7. These include the forward primers 5′-GCCACTGCAGCCGCCATAAT-3′ and 5′-GCCCCAGCTTTCCACCCATTAC-3′ and reverse primers 5′-CCCGAGGGCTGTGAGGAGTAA-3′ and 5′-CCCCCTCAGCCGCCGACTA-3′. Universal primers for RAG1 and S7 were detailed in López et al. [19] and Chow and Hazama [20], respectively. In addition, both forward and reverse internal primers were developed for S7. For RAG1, each 25 *μ*L PCR reaction consisted of 2 *μ*L of DNTPs, 2.5 *μ*L of 10X Taq buffer, 3 *μ*L of both forward and reverse primers, 10.375 *μ*L of dH_2_O, 1 *μ*L of Taq polymerase, or .125 *μ*L of HotStart* Taq* Polymerase (QIAGEN, Valencia, CA). Amplifications consisted of 35 cycles of an initial denaturation of 95°C for 15 minutes with an additional denaturation of 94°C for 40 seconds. This was followed by an annealing temperature of 55°C for 1 minute, an initial extension of 72°C for 90 seconds, and a final extension of 72°C for 5 minutes. Conditions for S7 were identical except the annealing temperature was set at 59°C. For the S7 intron, products that failed to amplify using the universal primers were reamplified using nested PCR reactions with the same conditions except for specific annealing temperatures as specified by the chemistry for the internal primers. Taxa failing to amplify with internal primers were cloned using the pGEM-T Easy Vector System kit (PROMEGA, Madison, WI) as outlined in Lang and Mayden [9]. PCR products were purified using QIAGEN gel extraction kits (QIAGEN, Valencia, CA). Sequencing was performed using a BigDye labeled dideoxy sequencing kit (BigDye) and visualized on an ABI 377 automated sequencer (Auburn University Molecular Genetics Instrumentation Facility, Auburn, AL) or an ABI 3700 (Macrogen Sequencing Facility, Seoul, South Korea). Both the heavy and light strands were sequenced for all samples and the sequences were aligned with Clustal X [21] with reference to the accompanying electropherograms. Some individuals contained heterozygote peaks in the RAG1 data and these heterozygote base pair positions were coded using standard degeneracy codes.

### 2.2. Phylogenetic Analyses

An incongruence-length difference analysis (ILD [22]) was performed with 1000 replicates to test for incongruence between the RAG1 and S7 data sets. Maximum parsimony (MP) analyses (MPA) were conducted in PAUP^*^4.0b10 [23]. All analyses consisted of a heuristic search model with 1000 random addition sequence replicates and TBR. Support for the parsimony analyses was generated using bootstrap analysis (BS) with 1000 bootstrap pseudo-replicates [18]. Bayesian analyses (BA) were conducted in Mr. Bayes 3.0b4 [24]. S7 intron all gaps were treated as missing data. The model of sequence evolution was determined using Modeltest v3.04 [25] with single partitions for each marker; the best-fit model for S7 was HKY + G and that for Rag1 was TrN + I + G. BA included four heated Markov chains using the default temperature setting. Log-likelihood scores were plotted against generation time to establish burn-in; trees prior to stationarity were discarded. Post-burn-in trees were used to develop the 50% majority rule consensus tree. Posterior probabilities (PP) were used as an indication of nodal support in BA.

## 3. Results and Discussion

**Figure 1:**
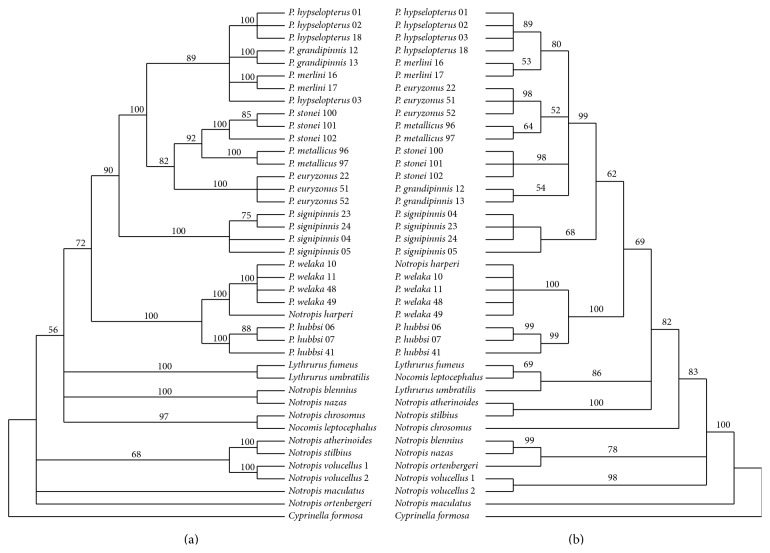
Inferred species relationships of species of* Pteronotropis* based on maximum parsimony analyses of RAG1 (a) and S7 (b). Nodal values indicate bootstrap support.

**Figure 2:**
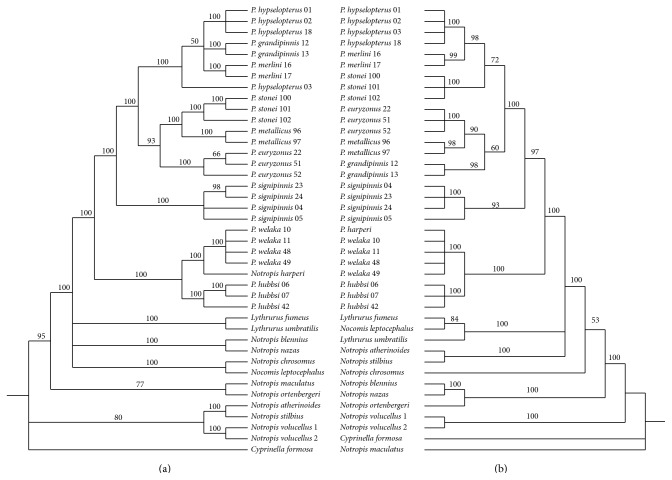
Inferred species relationships of species* Pteronotropis* based on Bayesian analyses of S7 (a) and Rag1 (b). Nodal values indicate posterior probabilities.

**Figure 3:**
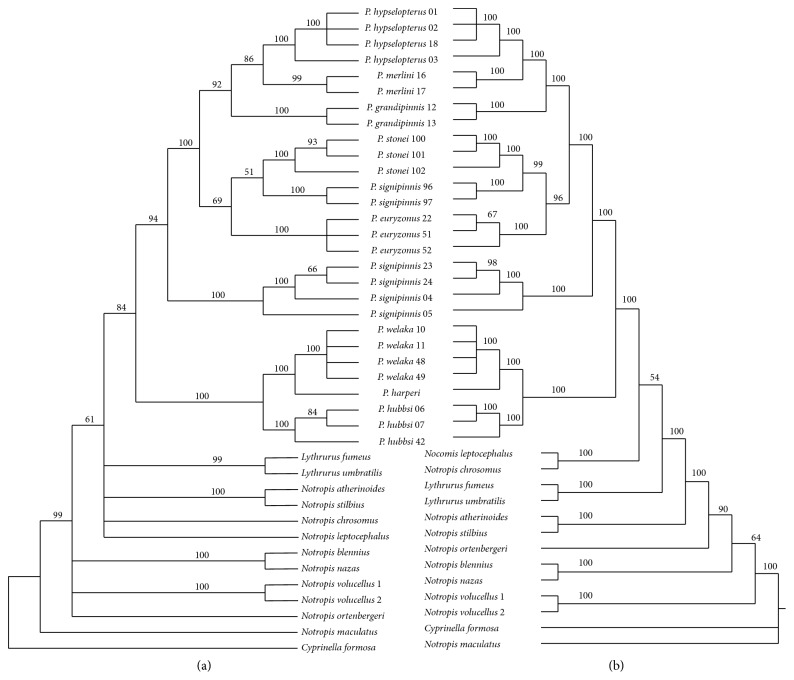
Inferred species relationships of species of* Pteronotropis* based on maximum parsimony and Bayesian analyses of combined Rag1 + S7 (a) and Rag1 + S7 (b), respectively. Nodal values indicate posterior probabilities.

As the ILD test was nonsignificant for heterogeneity between RAG1 and S7, the gene sequences were analyzed both individually and as a concatenated data set. MP analysis of the aligned 1001 bp of S7 (aligned sequence lengths ranged from 839 to 919 bp) yielded 245 bp parsimony informative sites (12.9%). Analyses of these data resulted in 90 equally parsimonious trees (Figure 1; length = 697, CI = 0.803, and RI = 0.875). The more conservative RAG1 sequences included 1521 bp with 151 bp sites (9.9%) being parsimony informative. MP analyses of RAG1 resulted in 46,668 equally parsimonious trees (Figure 1; length = 371 steps, CI = 0.658, and RI = 0.866). Individual BA analyses for each gene resulted in some variations in sister-group relationships but all were consistent and supported the monophyly of* Pteronotropis* (Figure 2). Both MPA and BA of the combined S7 + RAG1 data recovered identical topologies (Figure 3).

As in previous studies involving species of* Pteronotropis*, nuclear sequence variation, neither individual nor combined [5, 6], resolved* Pteronotropis* as a monophyletic group if* Notropis harperi* is excluded from the genus. Constraining* Pteronotropis* to be monophyletic in the S7 + RAG1 data set without* N. harperi* resulted in a significantly worse tree (1246 steps). In both BA and MPA,* Notropis harperi* is resolved as sister to* P. welaka* within the ingroup, a sister-group relationship with strong PP and BS support (Figures 1 and 2).* Pteronotropis hubbsi* is resolved as sister to this clade, also with strong PP and BS support. All three of these taxa (*P. hubbsi* (*P. welaka* +* N. harperi*)) are resolved as monophyletic and sister to the remaining species traditionally referred to as* Pteronotropis* (PP 95, bootstrap 75; Figure 2). The strong support for the monophyly of the (*P. hubbsi* (*P. welaka* +* N. harperi*)) clade (Figures 1 and 2) is logical as the three species are phenetically and ecologically similar. They possess aspects of similar body coloration in life when not in breeding condition and have similar habitat associations [5, 26, 27]. They are found in deep pools with ample aquatic vegetation and in areas where* P. welaka *and* N. harperi* are sympatric they are often taken syntopically in a sample (pers. obs.). The authors are unaware of any studies corroborating nest association in* N. harperi*, as observed in* P. welaka* and* P. hubbsi* [28–30]. In light of the relationships presented here and in Bufalino and Mayden [4, 5] and Hollingsworth et al. [10], studies of* N. harperi* may reveal ecological and behavioral synapomorphies.

In all analyses,* P. signipinnis* is resolved as sister to a clade of remaining species of* Pteronotropis* (Figures 1 and 2). In analyses of S7 and S7 + Rag1 data sets, the latter clade formed two clades: one inclusive of* P. hypselopterus*,* P. grandipinnis*, and* P. merlini* and the other inclusive of* P. euryzonus*,* P. stonei*, and* P. metallicus*. Resolution of the former clade was not complete in either Rag1 or S7 analyses, but both are fully consistent with the phylogeny recovered with the Rag1 + S7 data set. These relationships are in contrast to those hypothesized by Simons et al. [4] based on 12S and 16S ribosomal RNA sequences wherein* P. signipinnis* was resolved as sister to* P. welaka* +* P. hubbsi*. However, this latter study did not include all of the then or currently known species of* Pteronotropis*.

## 4. Conclusions

Given the consistent sister-group relationship between formerly recognized* Notropis harperi* and* Pteronotropis welaka*, the former species is herein referred to as* Pteronotropis*. Nuclear genes RAG1 and S7 support the long-standing question/hypothesis regarding the monophyly of* Pteronotropis* and provide new insight into the phylogenetic placement of* Pteronotropis harperi* and the basal-most relationships between the species groups (*P. hubbsi*,* P. welaka*, and* P. harperi*) relative to the remaining species of* Pteronotropis*. These relationships are also consistent with those presented by Bailey and Suttkus [13] using mitochondrial gene ND2. In recent years, the general trend in phylogenetics has been to place greater emphasis on the use of nuclear genes, largely because of issues associated with hybridization, intergradation, lineage sorting, and disagreement between gene and species trees [13]. While these nuclear genes have shown a greater ability to resolve relationships at supraspecific levels for this group with greater consistency and stronger branch support, the results presented herein illustrate the benefit in using nuclear genes. However, it is also true that mitochondrial genes have been extremely useful in phylogenetic resolutions [26, 27], and like nuclear genes they also vary in their degree of anagenesis and abilities to resolve trees at different levels of universality. While these and other nuclear genes used in the above-cited papers for Cypriniformes clearly display a reduced phylogenetic signal and are more limited in phylogenetic resolution for relationships of populations and species, they are essential for resolution of deeper nodes. This is to be expected as rates of mutation of many nuclear genes (especially protein coding) are generally not as high as that typically found in most mitochondrial genes.

## Figures and Tables

**(a):** 

Species and drainage	Stream, county, state	Catalogue number	S7	RAG1	Extraction
***Pteronotropis euryzonus***					
Chattahoochee R.	Maringo Cr., Russell, AL	UAIC 12229	KM048270	KJ634252	22
Chattahoochee R.	Snake Cr., Russell, AL	UAIC 10493	KM048276	KJ634258	51
Chattahoochee R.	Snake Cr., Russell, AL	UAIC 10493	KM048277	KJ634259	52
***Pteronotropis grandipinnis***					
Apalachicola R.	Irwin Mill Cr., Houston, AL	No voucher	KM048265	KJ634247	12
Apalachicola R.	Irwin Mill Cr., Houston, AL	No voucher	KM048266	KJ634248	13
***Pteronotropis hypselopterus***					
Mobile R.	Cedar Cr., Mobile, AL	UAIC 12730	KM048256	KJ634238	01
Mobile R.	Cedar Cr., Mobile, AL	UAIC 12730	KM048257	KJ634239	02
Mobile R.	Cedar Cr., Mobile, AL	UAIC 12730	KM048258	KJ634240	03
Alabama R.	Little Reedy Cr., AL	UAIC 14326	KM048269	KJ634251	18
***Pteronotropis hubbsi***					
Ouachita R.	Backwater pond, Ouachita, LA	UAIC 11928	KM048261	KJ634243	06
Ouachita R.	Backwater pond, Ouachita, LA	UAIC 11928	KM048262	KJ634244	07
Little R.	Little R., McCurtain, OK	UAIC 12053	KM048273	KJ634255	41
***Pteronotropis merlini***					
Pea R.	Clearwater Cr., Coffee, AL	No voucher	KM048267	KJ634249	16
Pea R.	Clearwater Cr., Coffee, AL	No voucher	KM048268	KJ634250	17
***Pteronotropis metallicus***					
Suwannee R.	Sampson R., Bradford, FL	UF 158855	KM048278	KJ634260	96
Suwannee R.	Sampson R., Bradford, FL	UF 158855	KM048279	KJ634261	97
***Pteronotropis signipinnis***					
Pascagoula R.	Beaverdam Cr., Forest, MS	UAIC 13416	KM048259	KJ634241	04
Pascagoula R.	Beaverdam Cr., Forest, MS	UAIC 13416	KM048260	KJ634242	05
Mobile R.	Cedar Cr., Mobile, AL	UAIC 12730	KM048271	KJ634253	23
Mobile R.	Cedar Cr., Mobile, AL	UAIC 12730	KM048272	KJ634254	24
***Pteronotropis stonei***					
N. Fork Edisto R.	Murphy Mill Cr., Calhoun, SC	SLUM 1121	KM048281	KJ634263	101
N. Fork Edisto R.	Murphy Mill Cr., Calhoun, SC	SLUM 1121	KM048280	KJ634262	100
Combahee R.	Savannah Cr., Colleton, SC	SLUM 1122	KM048282	KJ634264	102
***Pteronotropis welaka***					
Cahaba R.	Lightsey pond, Bibb, AL	UAIC 10391	KM048263	KJ634245	10
Cahaba R.	Lightsey pond, Bibb, AL	UAIC 10391	KM048264	KJ634246	11
Pearl R.	Lees Cr., Washington, LA	UAIC 12205	KM048274	KJ634256	48
Mobile Bay	Lees Cr., Washington, LA	UAIC 12205	KM048275	KJ634257	49
***Pteronotropis harperi***			GU134235	GU136332	

**(b):** 

Outgroup taxa (note that *Pteronotropis harperi* was also originally an outgroup species)		
Species	S7	RAG1
***Cyprinella formosa***	GU 134192	GU136293
***Lythrurus fumeus***	GU134222	GU136231
***Lythrurus umbratilis***	GU134223	GU136322
***Nocomis leptocephalus***	GU134236	GU136333
***Notropis asperifrons***	GU134231	GU136330
***Notropis atherinoides***	GU134232	EF452832
***Notropis blennius***	GU134234	GU136331
***Notropis leuciodus***	GU134237	GU136334
*Notropis maculatus *	GU134238	GU136335
*Notropis ortenburgeri *	GU134240	GU136337
***Notropis nazas***	GU134239	GU136336
***Notropis stilbius***	GU134241	GU136338
***Notropis volucellus*** 1	GU134242	GU136339
***Notropis volucellus*** 2	GU134243	GU136340
